# Hybrid expert system for robust detection of rare sequence signals: a computational proof-of-concept in host-dominated backgrounds

**DOI:** 10.3389/fmicb.2026.1821073

**Published:** 2026-04-07

**Authors:** Shenjie Wang, Liang Xie, Zhiyu Yan, Jinkai Ma, Ruidan Zhao

**Affiliations:** 1 School of Computer Science and Technology, Xi’an Jiaotong University, Xi’an, China; 2 Xi’an People’s Hospital (Xi’an Fourth Hospital), Xi’an, China

**Keywords:** ecosystem robustness, host-associated microbiome, hybrid expert-system, low-abundance signal detection, microbial symbiosis, sequencing artifact suppression

## Abstract

Deciphering microbial symbiosis in robust environmental and host-associated systems increasingly depends on the reliable recovery of weak biological signals from sequencing data dominated by non-target background. In practice, low-abundance symbiont-derived signatures are often obscured by background DNA, recurrent technical artifacts, and context-dependent false-positive calls, limiting the accuracy of downstream ecological and functional inference. Here, we present SymbioFilter, a hybrid expert system designed to improve the specificity and stability of rare-signal detection in host-associated microbiome sequencing data with substantial background noise. (i.e., overwhelming host-derived DNA, recurrent alignment errors, and sequencing artifacts). Specifically, this framework is intended for specialized usage cases where standard host-depletion strategies fail, such as capturing minor microbe-induced host somatic mutations or rare microbial homologs, which reflect subtle and intimate interactions between microbes and the host. SymbioFilter integrates three coordinated layers: (i)ensemble-based candidate detection, (ii)machine-learned background/noise discrimination using an XGBoost classifier, and (iii) rule-guided suppression of artifact-prone genomic regions using curated repetitive and blacklist annotations. Rather than relying on a single decision rule, the framework combines probabilistic classification with expert-defined constraints to preserve weak true signals while reducing recurrent false positives. This design specifically targets analytical failure modes common to host-dominated and low-input datasets, where precision is frequently compromised by rare-event noise. As a stringent proof-of-concept benchmark, we evaluated SymbioFilter in synthetic gradient spike-in datasets. Crucially, to ensure rigorous computational evaluation with an absolute ground truth—a standard that is currently unattainable in complex, real-world microbiome samples at ultra-low abundances—we utilized plasma cfDNA-like low-frequency conditions as a highly controlled, demanding proxy. Across gradient abundance levels, the framework consistently reduced false-positive inflation, improved agreement with the known ground truth, and maintained stable classification performance. Compared with a baseline pipeline and a widely used variant-calling workflow, SymbioFilter achieved lower mean squared error, stronger true-positive/true-negative balance, and consistently high precision-recall behavior, particularly under the most challenging low-abundance settings. Although validated here in a proxy benchmark environment, the computational principles of SymbioFilter address a broader class of sequencing problems central to microbial symbiosis research: identifying rare, functionally relevant biological signals in complex, noise-prone backgrounds. By providing a transferable, modular, and open computational strategy for robust signal recovery, SymbioFilter offers a useful methodological foundation for future studies of host-microbe interactions, resilient community assembly, and symbiosis-associated ecosystem stability. The code is freely available for academic use at https://github.com/hello-json/SymbioFilter.

## 1 Introduction

Microbial symbiosis is a major determinant of stability, resilience, and adaptive capacity in host-associated and environmental systems (Human Microbiome Project Consortium, 2012; Sommer and Bäckhed, 2013). Across gut, soil, aquatic, and other complex niches, microbial partners collectively modulate nutrient turnover, stress buffering, immune interactions, and recovery after disturbance (Shade et al., 2012; Widder et al., 2016). As sequencing-based profiling becomes increasingly central to symbiosis research (Quince et al., 2017), the ability to accurately detect weak but functionally relevant biological signals has emerged as a critical analytical bottleneck (Salter et al., 2014). In many realistic datasets, especially those derived from host-associated samples, target microbial or interaction-associated signals are sparse, highly heterogeneous, and easily obscured by dominant non-target background, making robust inference substantially more difficult than in idealized high-abundance settings (Eisenhofer et al., 2019).

A central challenge in this context is that rare sequence signals are disproportionately vulnerable to technical and analytical noise (Karstens et al., 2019; Davis et al., 2018). When target abundance is low, false-positive calls arising from recurrent artifacts, ambiguous alignment in repetitive or low-complexity regions, uneven coverage, and sample-specific background structure can overwhelm the true signal (Nayfach and Pollard, 2016). This problem is particularly acute in host-dominated sequencing contexts, where biologically meaningful low-abundance features may be masked by overwhelming host-derived material or confounded by recurrent background-associated patterns (Marotz et al., 2018). As a result, downstream estimates of signal burden, interaction intensity, or community-associated functional signatures may become inflated, unstable, or poorly reproducible, ultimately weakening ecological interpretation.

Despite rapid advances in microbial community analysis, many existing computational workflows remain optimized either for general-purpose variant detection or for abundance profiling under relatively cleaner signal conditions (Bolyen et al., 2019; Wood et al., 2019). Crucially, while standard metagenomic workflows typically rely on physical or *in silico* host depletion (i.e., discarding sequencing reads that align to the host genome), this straightforward strategy becomes fundamentally unsuitable in specific symbiotic edge cases. For instance, when targeting rare microbial sequences that share high homology with the host, or when investigating microbe-induced host somatic mosaicism and endosymbiotic integration, discarding host-aligned reads results in an irreversible loss of critical biological signals (Pleguezuelos-Manzano et al., 2020; Riley et al., 2013; Dunning Hotopp et al., 2007). In such scenarios, treating candidate detection, artifact suppression, and background discrimination as separate tasks often results in fragmented pipelines that do not sufficiently balance sensitivity and specificity when signals are weak (Beghini et al., 2021). Methods that prioritize sensitivity alone can accumulate large numbers of spurious positives, whereas overly conservative filtering may discard biologically relevant rare events (Ye et al., 2019; McIntyre et al., 2017).

To address this gap, we developed a hybrid expert-system for noise-aware detection of rare sequence signals in host-associated samples (Knights et al., 2011). The framework combines multi-source candidate aggregation, machine-learned discrimination of background-associated noise, and expert-guided suppression of artifact-prone genomic regions within a unified and modular architecture. Rather than relying on a single filtering rule, the method integrates probabilistic classification with interpretable domain constraints, thereby improving robustness in analytically difficult low-signal settings (Pasolli et al., 2016). This design is intended to support more reliable extraction of weak biological signals while minimizing false-positive inflation that can distort downstream profiling.

To rigorously validate the mathematical robustness of this algorithmic design, establishing an absolute “ground truth” is essential for accurately calculating false-positive and false-negative rates. Because complex, real-world microbiome datasets currently lack such absolute ground truth at ultra-low abundance margins (e.g., <1%), we utilized a stringent, plasma cfDNA-like low-frequency spike-in benchmark as a controlled computational proxy (Poore et al., 2020). While this specific benchmark utilizes human somatic variants rather than actual microbial communities, it provides a perfectly controlled, demanding environment that heavily recapitulates the key analytical challenges of host-associated microbial symbiosis studies: namely rare-signal recovery, noise-aware filtering, and stable quantification under strong background interference.

In this study, we demonstrate that the proposed framework improves rare-signal recovery across abundance gradients by reducing spurious detections, preserving balanced classification behavior, and maintaining stable quantitative performance under challenging low-input conditions. Through a unified combination of machine learning and expert-defined constraints, this work contributes an adaptable computational proof-of-concept for more reliable analysis of host-associated sequencing data and offers a practical methodological foundation for future studies of microbial interactions, resilient community function, and ecosystem robustness (Zhou and Gallins, 2019).

## 2 Materials and methods

### 2.1 Overview of the methods

**FIGURE 1:**
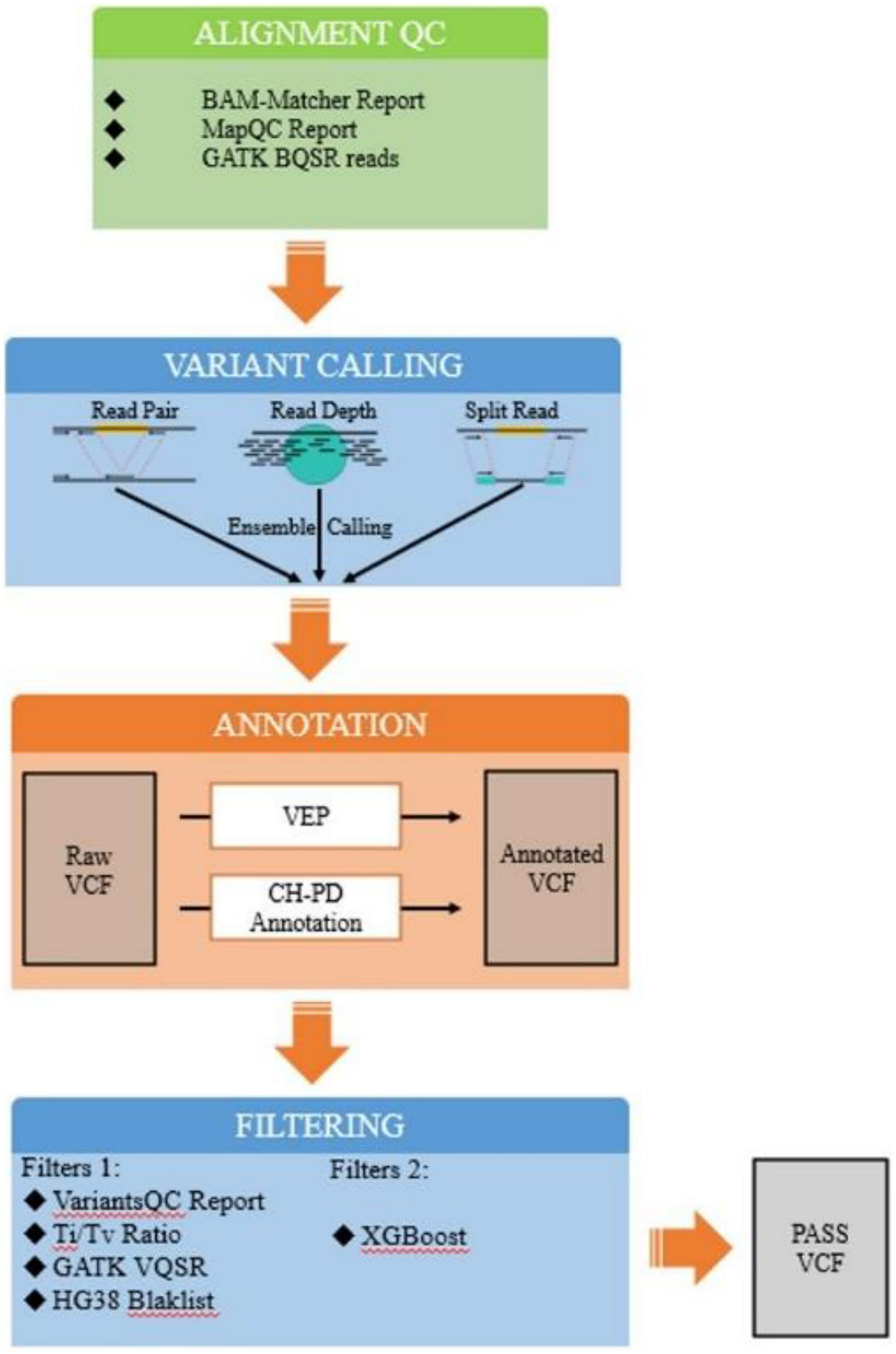
Overall architecture of the hybrid expert-system. The workflow begins with alignment quality assessment of raw sequencing reads, followed by ensemble-based candidate signal detection using multiple complementary calling strategies. Candidate events then enter a dual-track annotation stage, in which conventional functional or positional annotations are combined with a machine-learning-based background-risk assessment module. The annotated candidates are subsequently processed through a two-tier filtering layer composed of (i) expert-defined quality and region-aware exclusion rules and (ii) probabilistic classification for noise/background discrimination. High-confidence retained events are finally aggregated into sample-level signal burden estimates and detailed decision logs, enabling transparent and reproducible downstream analysis.

To support robust recovery of rare sequence signals in host-associated samples, we developed a modular hybrid expert-system that integrates candidate generation, multi-layer annotation, noise-aware filtering, and final burden-level quantification within a unified computational workflow. The framework is designed for analytical settings in which biologically relevant signals are sparse and embedded in strong background interference, a scenario frequently encountered in host-associated sequencing and particularly relevant to difficult microbial symbiosis profiling tasks involving low-abundance or weakly represented sequence features (Figure 1).

The overall workflow consists of five coordinated stages: (i) alignment-level quality control, (ii) ensemble-based candidate signal detection, (iii) dual-track annotation, (iv) two-tiered filtering combining expert-defined rules with machine-learned discrimination, and (v) sample-level quantification of retained high-confidence signals.

Rather than relying on a single threshold or a single caller, the framework explicitly separates signal discovery and noise suppression into distinct but connected stages. This design allows the method to maintain sensitivity for weak candidate events while reducing recurrent analytical artifacts that would otherwise inflate downstream interpretation. In the present study, the framework is evaluated in a plasma-like low-frequency proxy benchmark, which serves as a controlled testbed for rare-signal recovery under host-dominated background conditions.

### 2.2 Step-by-step implementation

#### 2.2.1 Alignment quality control

For the host-associated proxy benchmark used in this study, all input FASTQ reads were aligned to the hg38 reference genome using BWA-MEM with default parameters, followed by standard post-alignment processing in GATK4. Duplicate reads were marked, base quality score recalibration (BQSR) was performed, and alignment statistics were summarized using SAMtools and Picard. Quality control criteria included mapping rate (>95%), duplication rate (<15%), and acceptable insert size distribution. BAM-Matcher was additionally applied to flag potential sample swaps or contamination events. These procedures ensured that downstream rare-signal detection was performed on a technically reliable alignment set and reduced the impact of upstream sequencing noise on subsequent candidate identification.

#### 2.2.2 Ensemble candidate signal detection

To improve sensitivity and robustness in low-abundance settings, candidate sequence signals were identified using an ensemble strategy that integrates the outputs of multiple callers, including Mutect2, FreeBayes, and VarDict. This multi-caller design reduces dependence on any single algorithm and improves recovery of weak events that may be missed or inconsistently represented by an individual caller. Because each caller captures partially distinct signal characteristics, their results were merged into a consensus candidate set using a weighted voting framework that incorporates read-pair orientation, depth deviation, and breakpoint consistency. The resulting candidate pool provided a broader yet still controlled basis for downstream annotation and filtering in host-dominated, noise-prone samples.

#### 2.2.3 Candidate signal annotation

Each candidate event was annotated through two parallel tracks and then merged into a unified enriched record:

(1) The first track performed conventional functional and positional annotation using VEP (v105, Ensembl release), generating gene-level and transcript-level information, including variant consequence, amino acid substitution, and transcript identifiers. This annotation layer provided interpretable biological context for retained high-confidence events.
(2) The second track implemented a background-confounder prediction module based on an internally trained XGBoost classifier using a curated host-derived background mutation set (MSK-CH, 2023). The classifier leveraged features including variant allele frequency (VAF), read balance, strand bias, genomic context, and mutational recurrence across hematopoietic samples. Based on these features, each candidate was classified as background-likely, uncertain, or target-signal-likely. The two annotation streams were then merged to produce an enriched VCF containing structural, functional, and probabilistic metadata for downstream filtering.

#### 2.2.4 Noise-aware filtering

Annotated candidates were subjected to a two-tier filtering procedure designed to suppress recurrent artifacts while preserving low-abundance true signals.

The first tier consisted of rule-based filtering. This step incorporated multiple quality constraints, including depth, base quality, mapping quality, and strand bias derived from the VariantsQC report. Additional filtering was applied based on Ti/Tv ratio consistency, adjusted according to panel size and sample context, and GATK VQSR score thresholds were used to retain only high-confidence calls. To further reduce recurrent mapping and alignment artifacts, candidates located within problematic genomic regions were excluded, specifically those overlapping the hg38 ENCODE Blacklist and UCSC RepeatMasker regions.

The second tier consisted of machine learning-based background discrimination. Candidates retained after rule-based filtering were further scored by the XGBoost classifier. Only candidates with a predicted background probability below a predefined threshold (0.25, determined empirically through cross-validation) were retained in the final high-confidence set. This second filtering layer substantially improved specificity while preserving weak true signals that would otherwise be difficult to distinguish from recurrent host-derived background noise.

#### 2.2.5 Sample-level rare-signal burden quantification

After filtering, the retained high-confidence events were aggregated into a sample-level rare-signal burden metric. In the current benchmark implementation, retained non-synonymous SNVs and indels were counted for each sample and normalized by the effective genomic territory sequenced, typically 30–35 Mb for exome-capture panels. Synonymous, intergenic, and intronic variants were excluded by default, although the framework supports customizable inclusion criteria depending on study design and analytical goals. Final outputs included both the sample-level burden estimate and detailed variant-level decision logs, enabling full traceability of each retained or excluded event across the complete analytical workflow.

## 3 Results

### 3.1 Experimental setup

To rigorously evaluate the performance of the proposed framework under weak-signal, host-dominated conditions, we constructed a host-associated proxy benchmark using synthetic spike-in datasets that mimic realistic low-abundance sequencing scenarios. Rare target events were generated by introducing curated somatic mutations from International Cancer Genome Consortium (ICGC) tumor datasets into healthy donor cfDNA background data, thereby creating a controlled test environment with known ground truth labels and preserved background noise characteristics. This design enabled direct assessment of the framework’s ability to recover true low-frequency signals while suppressing recurrent background-associated artifacts.

To capture a broad range of analytically challenging conditions, six signal-abundance levels were evaluated: 0.5%, 1%, 8%, 15%, 25%, and 35%. These levels were selected to span ultra-low, low, intermediate, and relatively high signal fractions, allowing systematic characterization of performance across progressively less noisy settings. For each abundance level, 25 independent synthetic samples were generated. This replicate-based design preserved sample-to-sample variability and provided a robust basis for comparing stability, sensitivity, and specificity across the full abundance gradient.

Performance was evaluated at both the event level and the sample level. At the event level, because the true status of each spiked-in event was known, standard classification metrics were computed, including precision, recall, and F1-score. At the sample level, retained high-confidence events were aggregated into a rare-signal burden estimate, and quantitative accuracy was assessed by calculating the mean squared error (MSE) between the estimated burden and the known ground-truth burden derived from the curated spike-in set. This two-level evaluation strategy allowed us to determine not only whether the framework correctly classified rare events, but also whether these improvements translated into stable and accurate sample-level quantification.

Three analytical settings were compared in the benchmark. First, the proposed hybrid expert-system was evaluated as the primary method. Second, a baseline ensemble calling pipeline lacking background-confounder discrimination and region-aware artifact suppression was used to assess the contribution of the integrated filtering strategy. Third, GATK Mutect2 was included as a widely used reference caller for quantitative comparison in burden estimation. This design enabled separate examination of two key questions: whether the proposed framework reduces false-positive inflation relative to a minimally filtered ensemble workflow, and whether these improvements yield more faithful burden estimates than a commonly used single-caller approach.

All benchmark analyses were performed using the same alignment and preprocessing framework described in the section “2 Materials and methods” to ensure comparability across tools and conditions. By combining controlled spike-in truth, realistic host-background noise, multiple abundance levels, and replicate sampling, this experimental setup provided a stringent and reproducible benchmark for evaluating rare-signal recovery in host-associated samples and for testing the transferability of the proposed framework to robust microbial symbiosis profiling scenarios.

### 3.2 Performance evaluation

#### 3.2.1 Low-abundance signal recovery across the signal-abundance gradient

To assess the robustness of the proposed framework under progressively more challenging weak-signal conditions, we examined the distribution of retained high-confidence sequence signals across the six signal-abundance levels in the proxy benchmark (0.5%, 1%, 8%, 15%, 25%, and 35%). For each abundance level, signal counts from the proposed framework were compared with those from a baseline ensemble calling workflow that did not include background-confounder discrimination or region-aware artifact suppression. This comparison allowed direct evaluation of whether the integrated hybrid filtering strategy could reduce spurious signal inflation while preserving stable recovery of true low-abundance events.

**FIGURE 2:**
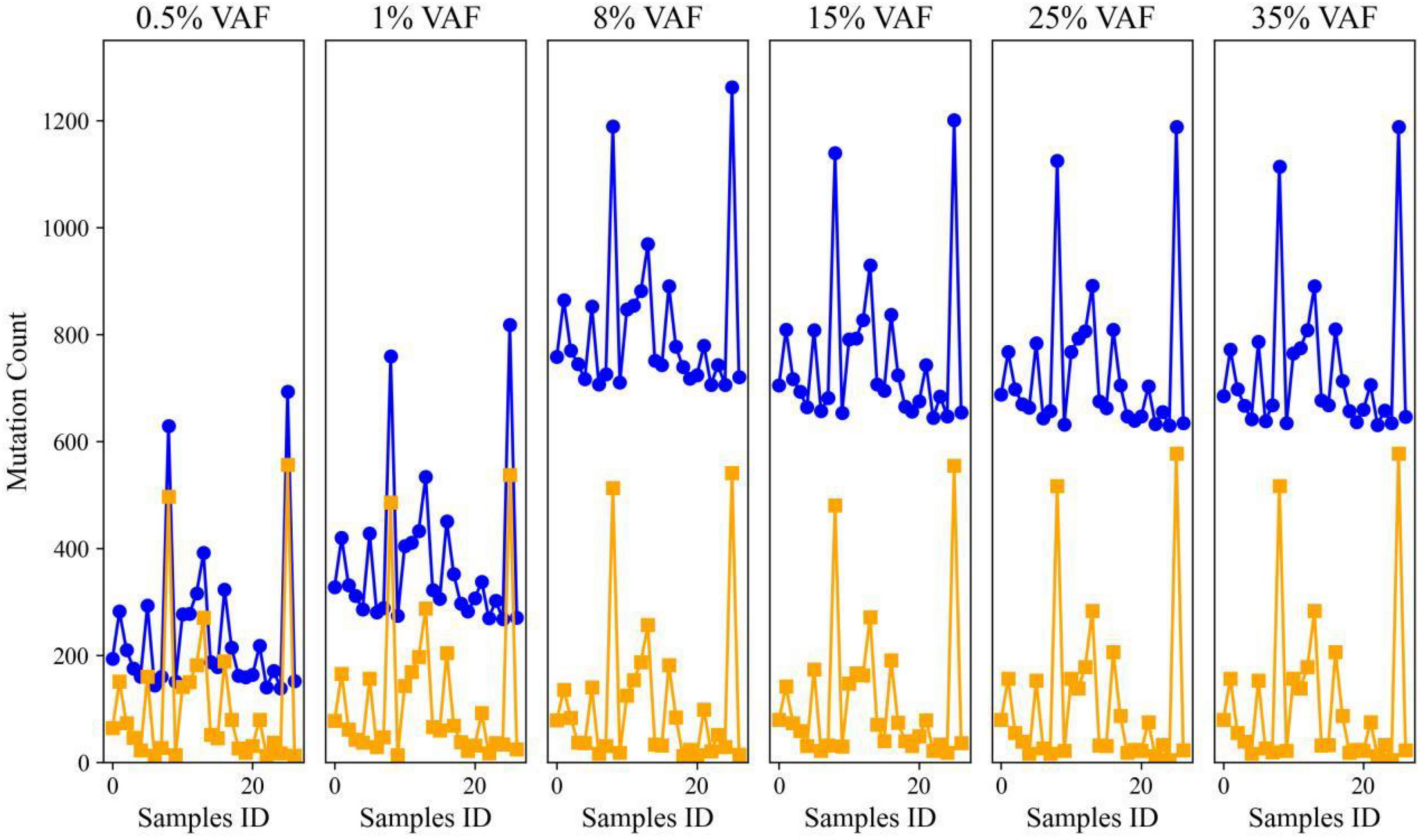
Distribution of retained high-confidence rare sequence signals across host-associated proxy samples with varying signal-abundance levels. Curated target events were introduced into healthy donor cfDNA sequencing backgrounds to generate a host-associated proxy benchmark spanning six signal-abundance levels (0.5%, 1%, 8%, 15%, 25%, and 35%). For each level, retained high-confidence signal counts were evaluated across 25 independent synthetic samples. Signal counts produced by the proposed hybrid expert-system are shown as orange squares, whereas counts from the baseline ensemble calling workflow without background-confounder discrimination or region-aware artifact suppression are shown as blue circles. The proposed framework consistently reduces spurious signal inflation, particularly under ultra-low and low signal-abundance conditions, resulting in more stable and biologically plausible retained-signal distributions. In contrast, the baseline workflow exhibits substantial count inflation and increased variability, especially at 0.5%–8%, indicating reduced specificity in host-dominated, noise-prone samples.

As shown in Figure 2, the proposed framework consistently produced lower and more stable retained-signal counts than the baseline workflow, with the largest differences observed in the most difficult low-abundance conditions (0.5%–8%). In these settings, the baseline method showed substantial count inflation and marked sample-to-sample variability, indicating that host-derived background confounders and artifact-prone regions contributed heavily to the raw candidate set when no dedicated suppression strategy was applied. By contrast, the proposed framework effectively constrained this inflation, yielding signal counts that were more consistent across replicates and more compatible with the expected ground-truth burden of the synthetic spike-in design.

The advantage of the proposed framework was especially pronounced at 0.5% and 1%, where rare-event detection is most vulnerable to noise. Under these ultra-low signal conditions, the baseline workflow substantially overcounted retained events, whereas the hybrid framework maintained a much tighter distribution, indicating stronger resistance to false-positive accumulation. Importantly, this suppression of overcounting did not lead to evidence of excessive signal loss at higher abundance levels. In the 15%–35% range, retained-signal counts remained stable and aligned with the expected trend across the gradient, supporting the view that the framework improves specificity in noisy samples without sacrificing practical sensitivity when signal quality improves.

Taken together, these results demonstrate that the combination of learned background discrimination and region-aware artifact filtering substantially improves rare-signal recovery in host-dominated sequencing contexts. By limiting spurious calls at the low-abundance end of the gradient while preserving stable behavior at moderate and high abundance levels, the framework provides a more reliable foundation for downstream sample-level burden estimation and for robust profiling of subtle biological signals in complex host-associated samples.

#### 3.2.2 Superior accuracy of sample-level rare-signal burden estimation across signal-abundance levels

To further evaluate whether improved event-level filtering translates into more accurate sample-level quantification, we compared the proposed framework with GATK Mutect2 using the host-associated proxy benchmark across six signal-abundance levels (0.5%, 1%, 8%, 15%, 25%, and 35%). For each abundance level, the retained high-confidence events were aggregated into a sample-level rare-signal burden estimate, and quantitative accuracy was assessed by calculating the mean squared error (MSE) between the estimated burden and the known ground-truth burden derived from the curated spike-in event set.

**FIGURE 3:**
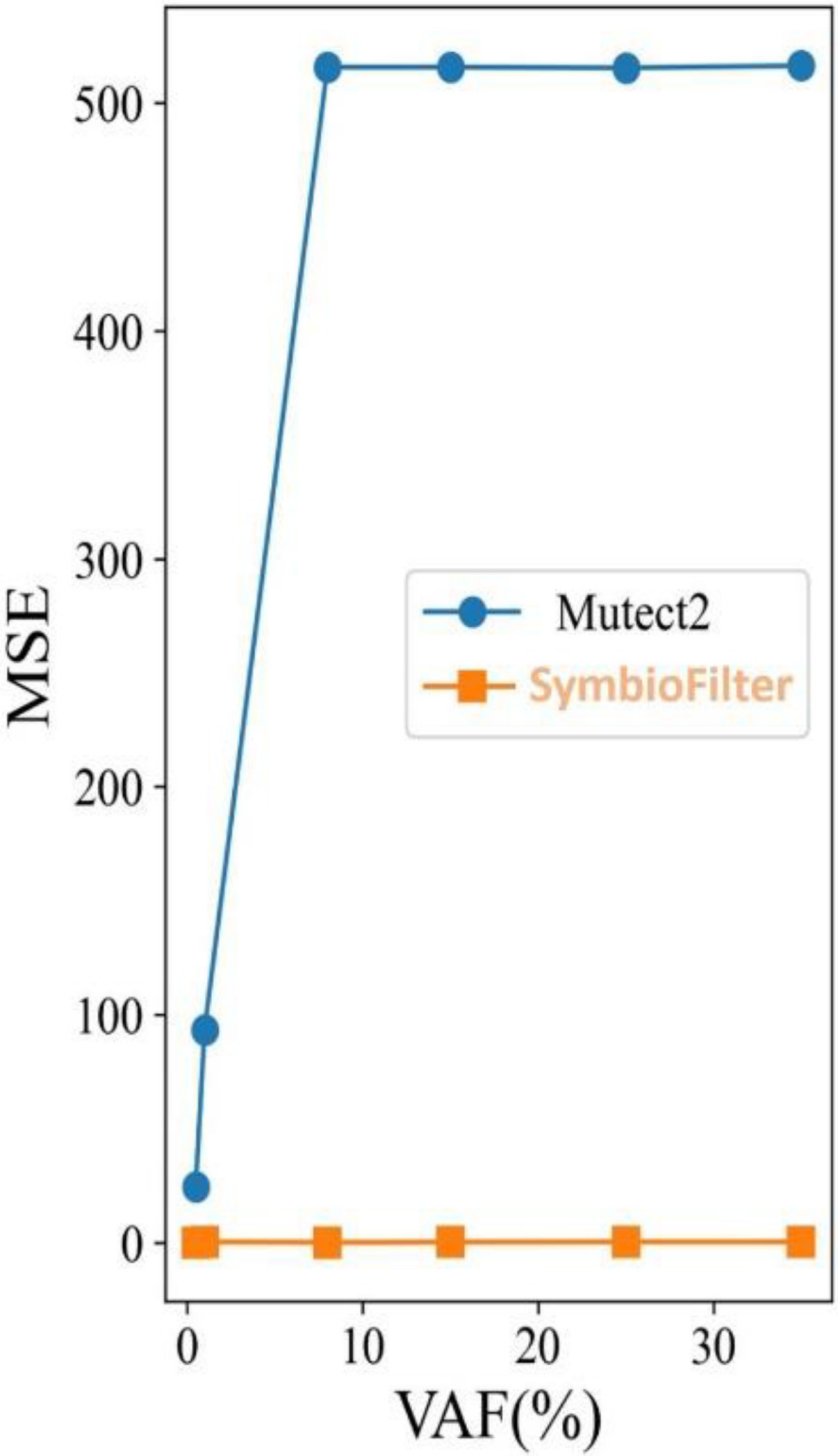
Comparison of sample-level rare-signal burden estimation accuracy between the proposed framework and GATK Mutect2 across six signal-abundance levels. Mean squared error (MSE) values were calculated between the estimated sample-level rare-signal burden and the known ground-truth burden in synthetic host-associated proxy datasets spanning six signal-abundance levels (0.5%, 1%, 8%, 15%, 25%, and 35%). The proposed framework is shown as orange squares, and GATK Mutect2 is shown as blue circles. The proposed framework maintains near-zero MSE across the full abundance gradient, indicating consistently accurate quantification of retained true signals even under ultra-low abundance conditions. In contrast, Mutect2 exhibits marked error inflation at low abundance levels, particularly at 0.5% and 1%, where MSE values exceed 500, reflecting substantial overestimation caused by false-positive accumulation in noisy host-dominated backgrounds. These results demonstrate the superior quantitative robustness of the proposed framework for burden estimation in weak-signal sequencing settings.

As shown in Figure 3, the proposed framework maintained consistently low MSE values across the full signal-abundance gradient, with near-zero deviation from the ground truth even under the most challenging ultra-low abundance conditions. This result indicates that the framework not only improves event-level specificity, but also preserves quantitative fidelity when translating retained events into a sample-level burden metric. Across all abundance levels, burden estimates remained tightly aligned with expected values, demonstrating stable performance in the presence of strong host-background interference.

In contrast, Mutect2 exhibited substantially elevated MSE values, particularly at 0.5% and 1%, where error inflation was most pronounced. Under these ultra-low abundance conditions, the quantitative deviation exceeded 500 MSE, indicating severe overestimation of the sample-level burden. This pattern is consistent with the accumulation of false-positive calls in noisy, low-frequency settings, where weak true signals are easily obscured by recurrent background-associated confounders and artifact-prone regions when no dedicated multi-layer suppression strategy is applied. Even as signal abundance increased, the error profile of Mutect2 remained markedly higher than that of the proposed framework, indicating limited robustness across the gradient benchmark.

These findings demonstrate that the gain in variant-level filtering quality is not merely cosmetic, but directly improves downstream quantitative interpretation. By preventing artificial inflation of retained-event counts and preserving closer agreement with the known truth set, the proposed framework provides a substantially more reliable estimate of sample-level rare-signal burden than a conventional single-caller workflow. This level of quantitative stability is especially important for host-associated sequencing analyses in which subtle abundance shifts may carry biological significance but are easily distorted by technical noise.

#### 3.2.3 Consistent classification performance across variable signal-abundance levels

To further assess the reliability of the proposed framework in distinguishing true retained signals from background-associated confounders, we analyzed classification outcomes across synthetic host-associated proxy samples spanning six signal-abundance levels (0.5%, 1%, 8%, 15%, 25%, and 35%). Because ground-truth labels were known for all introduced and background events, each retained or excluded event could be directly categorized as a true positive (TP), true negative (TN), false positive (FP), or false negative (FN), enabling explicit evaluation of filtering behavior under progressively different signal conditions.

For each abundance level, the normalized frequencies of TP, TN, FP, and FN outcomes were calculated across all test samples and visualized as heatmaps. In these heatmaps, rows represent individual synthetic samples and columns represent the four classification categories. Higher intensity in the TP and TN columns indicates strong recovery of true signals together with effective exclusion of background noise, whereas lower intensity in the FP and FN columns indicates suppression of spurious calls and limited loss of true events. This representation provides a sample-resolved view of classification balance rather than a single aggregate summary metric.

**FIGURE 4:**
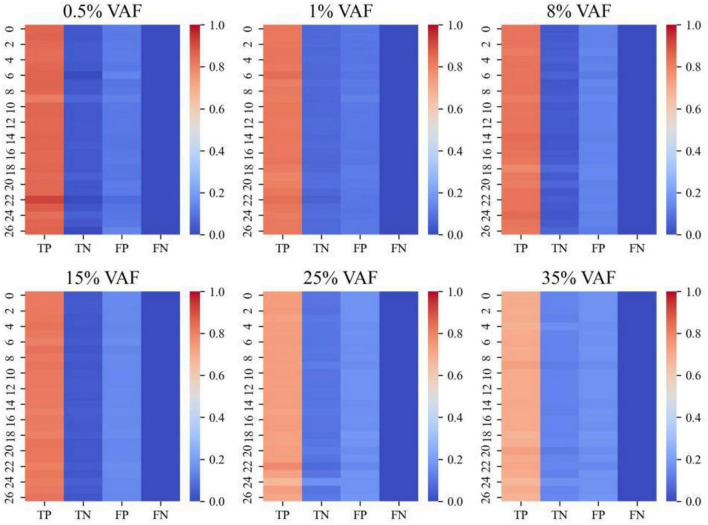
Heatmaps of classification outcomes for the proposed framework across six signal-abundance levels in host-associated proxy samples. Each panel corresponds to a synthetic host-associated proxy dataset with a specific signal-abundance level (0.5%, 1%, 8%, 15%, 25%, and 35%). Within each panel, rows represent individual synthetic samples, and columns correspond to four classification categories: true positives (TP), true negatives (TN), false positives (FP), and false negatives (FN). Color intensity indicates the normalized proportion of each classification outcome per sample. Across all signal-abundance levels, the proposed framework maintains strong TP and TN performance while effectively suppressing FP and FN rates. This pattern is preserved even under ultra-low abundance conditions, indicating stable and discriminative rare-signal filtering in host-dominated, noise-prone sequencing contexts.

As shown in Figure 4, the proposed framework maintains consistently strong TP and TN performance across the full signal-abundance gradient. Even under the most challenging ultra-low abundance conditions (0.5% and 1%), the method preserves a high proportion of correct classifications while keeping FP levels low. This pattern indicates that the framework remains discriminative in highly noisy host-dominated backgrounds, where false-positive inflation would otherwise be expected to increase substantially. As signal abundance rises beyond 8%, the TP component becomes further strengthened, while FP and FN signals remain stably suppressed.

Importantly, the FN proportion does not show a marked increase at the lowest abundance levels. This suggests that the framework achieves improved specificity without becoming overly conservative, thereby avoiding the common trade-off in which aggressive filtering reduces false positives at the cost of excessive true-signal loss. Instead, the heatmap pattern supports a balanced classification profile in which weak but relevant signals are retained while recurrent background-associated events are effectively excluded.

Together, these results demonstrate that the proposed framework delivers stable and well-balanced classification performance across a wide range of signal-abundance conditions. By simultaneously maintaining strong TP/TN behavior and suppressing FP/FN rates, the framework provides a robust basis for downstream rare-signal burden estimation and for reliable interpretation of subtle biological signals in complex host-associated sequencing data.

#### 3.2.4 Stable precision-recall performance across the signal-abundance gradient

To provide an integrated view of event-level classification quality across all signal-abundance conditions, precision and recall were jointly evaluated for each synthetic host-associated proxy sample and visualized in a precision-recall space. For every sample, precision was calculated from the proportion of retained events that matched the ground-truth introduced signals, whereas recall was calculated from the proportion of ground-truth signals successfully recovered after filtering. The harmonic mean of precision and recall was represented by background F1-score contours, enabling direct visualization of the balance between sensitivity and specificity across the full benchmark.

**FIGURE 5:**
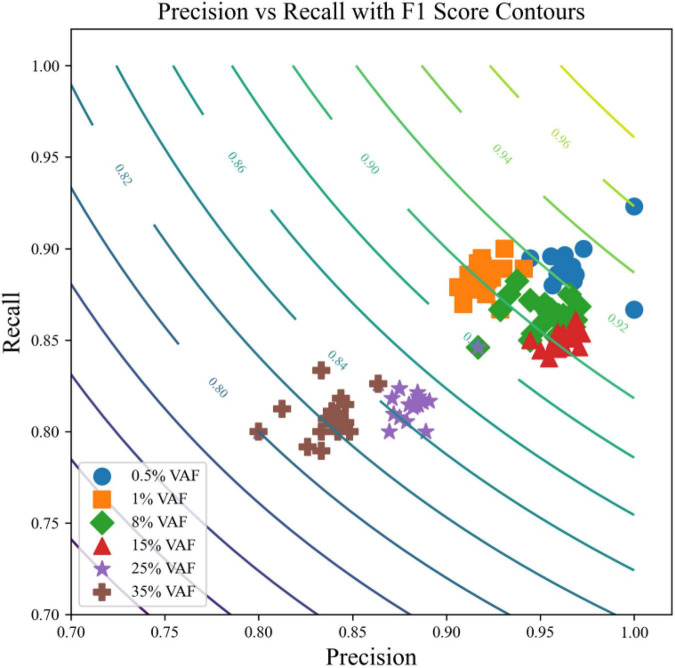
Precision-recall distribution of the proposed framework across six signal-abundance levels with F1-score contours. Each point represents one synthetic host-associated proxy sample, positioned according to its event-level precision and recall. Marker color and shape denote the six signal-abundance levels (0.5%, 1%, 8%, 15%, 25%, and 35%). Background isocurves indicate F1 scores, representing the harmonic mean of precision and recall. Samples are tightly clustered near the upper-right corner of the plot, showing that the framework maintains simultaneously high precision and high recall across nearly all abundance levels. Most samples fall above the F1 = 0.90 contour, indicating strong overall classification quality, including under ultra-low abundance conditions. A small subset of higher-abundance samples shows a modest reduction in precision, but overall F1 values remain high, demonstrating stable and well-balanced rare-signal filtering performance in host-dominated, noise-prone sequencing settings.

As shown in Figure 5, samples from all six signal-abundance levels cluster tightly in the upper-right region of the precision-recall plot, indicating that the framework maintains both high precision and high recall across a broad range of weak-signal conditions. Most samples lie above the F1 = 0.90 contour, demonstrating consistently strong overall classification quality. This result confirms that the framework does not merely suppress false positives, but does so while preserving substantial recovery of true low-abundance signals.

Notably, classification performance remains stable even at the most challenging ultra-low abundance levels (0.5% and 1%), where many analytical workflows typically experience a marked decline in either precision or recall. In contrast, the samples in this benchmark remain tightly grouped with limited dispersion, indicating low between-sample variability and strong resistance to sequencing noise under host-dominated background conditions. This compact distribution across replicates further supports the robustness of the framework in scenarios where target signals are sparse and easily confounded by recurrent background-associated artifacts.

At higher signal-abundance levels (25% and 35%), a modest reduction in precision is observed for a small subset of samples, consistent with occasional retention of residual non-target events after filtering. However, these shifts are limited in magnitude and do not substantially compromise the overall balance of precision and recall, as nearly all samples remain above F1 = 0.85. This pattern indicates that the framework sustains a favorable trade-off between sensitivity and specificity across both low- and high-abundance conditions, without showing evidence of catastrophic failure in any region of the gradient benchmark.

Together, these results demonstrate that the proposed framework achieves a stable and well-balanced classification profile across a wide dynamic range of signal-abundance levels. By maintaining tightly clustered high-quality precision-recall behavior in weak-signal settings, the framework provides strong support for its use in robust rare-signal recovery and downstream burden estimation in complex host-associated sequencing datasets.

## 4 Discussion

The present study demonstrates that a hybrid expert-system can substantially improve the recovery of rare sequence signals in host-associated, noise-prone sequencing data by explicitly integrating candidate aggregation, probabilistic background discrimination, and region-aware artifact suppression within a single analytical framework. Across the host-associated proxy benchmark, the framework showed stable performance over a wide signal-abundance gradient, reduced spurious signal inflation at the lowest abundance levels, preserved balanced classification behavior, and delivered markedly improved sample-level burden estimates relative to conventional comparison workflows. These gains are consistent with the underlying design of the method, which combines ensemble calling, dual-track annotation, rule-based exclusion, and machine learning-based filtering rather than relying on a single decision layer.

A central implication of these results is that weak-signal profiling in host-associated systems benefits from treating false-positive control as a primary design objective rather than as a downstream cleanup step. In low-abundance sequencing contexts, rare but biologically meaningful events are often confounded by recurrent background-associated signals, alignment ambiguity, and region-specific artifacts. Under such conditions, conventional pipelines may retain excessive non-target events, leading to inflated counts and unstable downstream interpretation. The current results show that explicitly modeling these confounding sources can improve both event-level and sample-level robustness, especially at the lowest signal fractions, where analytical failure is most likely to occur.

This property is particularly relevant to microbial symbiosis profiling in host-associated environments. Many symbiosis-linked sequencing questions involve detecting weak, sparse, or transient signatures against a dominant host-derived background, including low-abundance microbial sequence features, subtle interaction-associated markers, or functionally important signals present only in a subset of the sampled community. Although the present benchmark does not directly analyze real microbiome or symbiotic community datasets, it captures a key computational challenge shared by such studies: the need to distinguish weak true signals from pervasive non-target background in a reproducible and quantitatively stable manner. In this sense, the framework should be viewed as a transferable computational strategy for robust signal recovery in host-associated microbiome analysis rather than as a domain-specific solver restricted to the proxy benchmark used here. The study was explicitly built around low-frequency spike-in datasets and evaluated using precision, recall, F1-score, and MSE under a controlled gradient design, which supports this broader methodological interpretation.

An important strength of the framework is its modularity. Because candidate generation, annotation, rule-based filtering, and probabilistic discrimination are separated into distinct but connected stages, the pipeline can be adapted to other sequencing scenarios without requiring a complete redesign. In principle, the background-risk classifier can be retrained on alternative confounder classes, the region-aware masking layer can be replaced with reference resources appropriate to a different host or experimental system, and the final burden calculation can be re-parameterized for study-specific definitions of retained signal. This modular architecture increases the likelihood that the framework can be extended from the present host-reference proxy benchmark to broader applications in host-associated microbial profiling, ecological monitoring, or other rare-event detection tasks in complex biological mixtures.

Another notable strength is that the observed performance gains are not limited to a single metric. The framework reduced count inflation in the low-abundance range, improved quantitative agreement with ground truth in the sample-level burden analysis, maintained strong true-positive and true-negative behavior in the classification heatmaps, and preserved tightly clustered high-quality precision-recall profiles across the benchmark. This convergence across complementary evaluation criteria indicates that the method is not merely tuned to optimize one summary statistic, but instead improves the overall balance between sensitivity and specificity in analytically difficult settings. Such consistency is especially important for host-associated symbiosis studies, in which small analytical distortions can propagate into misleading biological conclusions when rare signals are used to infer community interactions, resilience thresholds, or disturbance responses.

At the same time, several limitations regarding the scope of this study must be acknowledged. First, and most importantly, the present evaluation was conducted in a synthetic host-associated proxy benchmark utilizing human genomic data and somatic mutations, rather than in genuine microbial symbiosis datasets. We purposefully selected this proxy environment because evaluating rare-signal recovery at ultra-low frequencies (e.g.,<1%) requires a mathematically absolute ground truth to accurately compute precision, recall, and mean squared error (MSE). Currently, real-world microbiome datasets lack such precise ground truth at these extreme low-abundance margins, making it difficult to confidently distinguish algorithmic false positives from genuine, ultra-rare microbial signals. Therefore, our results should be strictly interpreted as a computational proof-of-concept demonstrating how explicitly modeling background noise can improve weak-signal recovery in host-dominated environments, rather than a direct biological validation of a microbiome workflow.

Second, the benchmark implementation uses a human host reference and human-specific artifact resources (e.g., the HG38 blacklist). While these are suitable for establishing the statistical robustness of the proposed hybrid architecture, they are not directly transferable to specific microbiome applications without extensive reconfiguration. Third, the probabilistic background model was trained on a specific confounder class in the benchmark system, meaning its current decision boundary is implementation-specific. These constraints emphasize that SymbioFilter is currently a methodological foundation, and its direct deployment in metagenomic studies will require condition-specific optimization.

Future work should therefore focus on three directions. First, the framework should be revalidated in real host-associated microbiome datasets, particularly samples with strong host background and independently supported truth surrogates, to determine how well the current performance profile generalizes beyond synthetic spike-in data. Second, the background-risk discrimination module should be retrained using microbiome-relevant confounder sets, including host-derived off-target signals, recurrent contamination signatures, and taxa-specific alignment artifacts. Third, the current burden-oriented output could be expanded toward richer downstream representations, such as interaction-aware signal classes, taxa-stratified burden summaries, or multi-modal integration with metabolomic and ecological metadata. These extensions would move the framework from a robust rare-signal recovery engine toward a broader computational platform for studying symbiosis-associated system robustness.

In summary, this study supports the view that robust microbial symbiosis profiling in host-associated systems may require computational frameworks that explicitly model low-abundance signal detection as a structured noise-discrimination problem. By unifying ensemble candidate discovery, context-aware annotation, expert-rule filtering, and probabilistic background suppression, the proposed framework provides a practical and extensible foundation for extracting weak but informative biological signals from complex sequencing backgrounds. Although further validation in real symbiosis datasets remains necessary, the results presented here establish a strong methodological case for transferring this design strategy to future studies of host-microbe interactions, resilient community assembly, and ecosystem robustness in challenging sequencing environments.

## 5 Conclusion

In summary, the present study establishes a hybrid expert-system for noise-aware recovery of rare sequence signals in host-associated samples and demonstrates that explicit integration of ensemble candidate detection, probabilistic background discrimination, and region-aware artifact suppression can substantially improve analytical robustness in weak-signal sequencing settings. By addressing low signal-to-noise conditions as a structured noise-discrimination problem, the framework provides a practical and modular strategy for extracting biologically meaningful signals from complex, host-dominated backgrounds.

Across the host-associated proxy benchmark, the framework consistently reduced spurious signal inflation, maintained stable classification quality across signal-abundance levels as low as 0.5%, and achieved markedly improved sample-level burden estimation accuracy relative to conventional comparison workflows. Its ability to preserve true low-abundance signals while effectively removing background-associated confounders supports its utility as a robust computational solution for rare-signal profiling under analytically challenging conditions.

Although validated here in a synthetic host-associated proxy system, the computational principles of this framework are directly relevant to broader host-associated microbiome and microbial symbiosis studies, where weak but functionally important sequence signals are often embedded within strong non-target background. By providing a transferable, extensible, and quantitatively stable analytical design, this work offers a useful methodological foundation for future research on robust microbial symbiosis profiling, host-microbe interaction analysis, and ecosystem resilience in complex sequencing environments.

## Data Availability

The original contributions presented in this study are included in this article/supplementary materials, further inquiries can be directed to the corresponding author.
